# Improving Hunted Wild Boar Carcass Hygiene: Roles of Different Factors Involved in the Harvest Phase

**DOI:** 10.3390/foods10071548

**Published:** 2021-07-04

**Authors:** David Ranucci, Rossana Roila, Andrea Onofri, Fausto Cambiotti, Sara Primavilla, Dino Miraglia, Egon Andoni, Alessandro Di Cerbo, Raffaella Branciari

**Affiliations:** 1 Department of Veterinary Medicine, University of Perugia, Via San Costanzo 4, 06121 Perugia, Italy; david.ranucci@unipg.it (D.R.); dino.miraglia@unipg.it (D.M.); raffaella.branciari@unipg.it (R.B.); 2 Department of Agriculture, Food and Environmental Sciences, University of Perugia, Borgo XX Giugno 74, 06126 Perugia, Italy; andrea.onofri@unipg.it; 3 Health Department Umbria 1-Alto Chiascio, Via Cavour 38, 06024 Gubbio (PG), Italy; fausto.cambiotti@uslumbria1.it; 4 Istituto Zooprofilattico Sperimentale dell’ Umbria and Marche ‘T. Rosati’, Via Salvemini 1, 06126 Perugia, Italy; s.primavilla@izsum.it; 5 Faculty of Veterinary Medicine, Universiteti Bujqësor i Tiranës, Kodër Kamëz, SH1, 1000 Tiranë, Albania; eandoni@ubt.edu.al; 6 School of Biosciences and Veterinary Medicine, University of Camerino, 62024 Matelica, Italy; alessandro.dicerbo@unicam.it

**Keywords:** game meat, food safety, process hygiene criteria, Salmonella

## Abstract

Game meat production strongly differs from that of other meats, as peculiar factors present in the field and in the steps prior to transfer to a game-handling establishment can influence the hygiene of the carcasses and, therefore, of the meat. The effects of such factors were considered in hunted wild boars based on the main hygienic criteria adopted in meat processing. Environmental, animal, and hunting conditions were studied during two selective hunting seasons in Central Italy. A total of 120 hunted wild boar carcasses were sampled after the skinning process and analyzed for aerobic colony count, *Enterobacteriaceae* count, and *Salmonella* spp. isolation. The calculated mean values for aerobic colony and *Enterobacteriaceae* counts were 3.66 and 2.05 CFU/cm^2^, respectively, in line with the limits set for the meat of other ungulates by EU legislation. *Salmonella* spp. showed a prevalence of 2.5% (IC 95%: 1.72–3.27%). Statistical analysis of the data performed with the AIC criterion showed that the main parameter to consider for improving the hygienic level of carcasses is to reduce the time in the refrigerator before skinning, followed by hunting on cold days (<10 °C) without rain, hunting animals <60 kg, and reducing the time between shooting and evisceration.

## 1. Introduction

Eating game meat is common worldwide and, apart from food security issues linked with specific territories where hunting is a relevant and resilient practice [1,2], is increasing in Europe and other industrialized regions [3,4]. This type of meat is in line with modern consumers’ demand for its unique characteristics, not only from a nutritional and sensory point of view, but also in relation to sustainability, the avoidance of chemicals, and animal welfare [5,6,7,8,9].

Wild boar is the most widespread species in Europe, due to its adaptability and fertility [10,11], along with the increased availability of forest and abandoned rural areas where they can live [6]. The increased wild boar population is generating problems in some countries due to expanding contact with urban environments, such as damage to crop production, collisions with vehicles, and possible spread of zoonoses [11]. The population dynamics are generally controlled by hunters; therefore, the availability of wild boar meat is growing [3], and this could become a relevant part of regional economies not only for fresh meat production but also several typical meat products appreciated by consumers [12]. For this reason, certified game meat production chains following a “forest to fork” approach are desirable to obtain high-quality meat to fulfill consumer demand [13].

Nonetheless, some gaps can be found in the hygienic production of such game meat, for which the harvest process is generally conducted in a wild environment, not always ideal for meat production. In such an environment, the animals can be more easily contaminated during hunting and related practices, or the time or conditions to process them quickly and properly varies [14]. Furthermore, the production chain is peculiar and organized in different steps that involve the responsibility of different individuals who must guarantee meat safety and optimal hygienic quality [15]. In fact, parts of the production chain are under hunters’ control, while others are managed by the owners of collection centers (usually hunters) and/or game-handling establishments, and only this final step is under official control by veterinarian officers and follows the same rules of “farmed” meat chain production. This peculiar chain must be properly set up in the harvest phase to avoid the possibility that a gap in the correct implementation of hygienic procedures in one step could lead to an increased health risk for consumers or reduce the shelf life of the product due to relevant bacterial growth [14].

Different authors have reported the hygienic characteristics of wild boar meat [14,16,17,18]; however, a multifactorial evaluation of the combined effects of harvest factors on the main microbiological criteria adopted for process hygiene and meat safety has been rarely reported [19,20].

The aim of the paper is, therefore, to give insight into the main factors associated with hunters with regard to the harvest process affecting the hygiene of hunted wild boar carcasses. The results can point out valuable tools to provide common practices and easy strategies for hunters to adopt in order to improve wild boar carcass hygiene in valuable commercial chains for the game meat market.

## 2. Materials and Methods

### 2.1. Hunting and Carcass Sampling

The trial was conducted on 120 wild boars collected during 2 hunting seasons (2018 and 2019), performed to control their population, in the Umbria region (Central Italy). In this control program, 2 hunting systems were considered: “aspetto” (or “still” hunting), without dogs, just waiting for wild boar to pass in front of specific hidden shooting points, and “girata,” performed with dogs kept on leashes that reveal the presence of wild boar and leave the hunters to properly check and shoot them. Even though the girata method is generally used by 4 or more hunters, a single hunter was enrolled for the 2 seasons (other than the dog handlers) and was asked to complete a specific survey questionnaire for each hunted animal, such as that reported by Branciari et al. [21] but implemented with the adopted hunting technique, and to define specific factors potentially related to the hygienic condition of the carcasses during harvesting. The distribution of samples according to considered factors is reported in Table 1. Hunted animals were accurately evaluated for their health status before and after shooting, and during the hunt a rifled gun with no-lead ammunition (7 mm) was used. A specific protocol was provided to the hunter: avoid multiple shots and shoot in the abdomen, bleed the animals properly and quickly on the field, and trace the carcasses with single plastic clamps. 

The collected hunted wild boars were properly processed at a nearby collection center (2 km from the hunting area, at Azienda Faunistico Venatoria Serra Brunamonti, Gubbio PG, Italy), where the carcasses were weighed, properly eviscerated, and refrigerated without skinning at 5 ± 1 °C. After 2, 4, or 6 days at the collection center (authorized by EU Regulation 852/2004 [22]), carcasses were hygienically transported under refrigeration to the local game-handling establishment, where skinning was promptly performed (as set by EC Regulation 853/2004 [23]).

**Figure 1:**
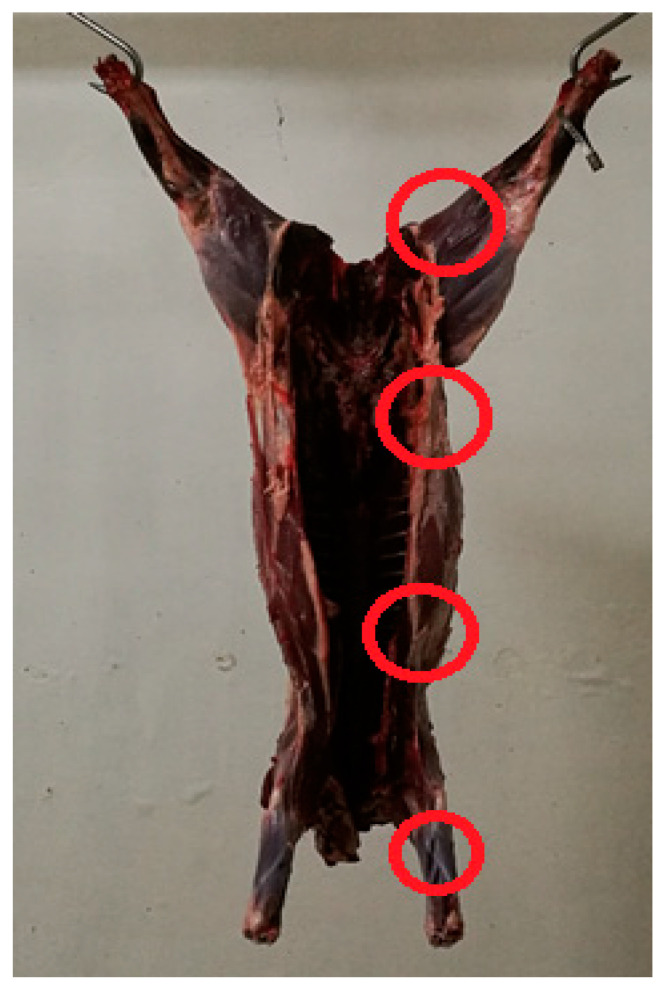
Sampling points on wild boar carcass selected for microbial determination.

After skinning at the game-handling establishment, 4 samples of 5 cm^2^ each (1 × 5 cm) were collected from each carcass by the reference excision method on the surface of 4 specific parts that could be considered the most prone to contamination (rump, flank, brisket, and foreleg; Figure 1) [21], pooled in sterile stomacher bags, placed in refrigerated containers, and quickly moved to the microbiology laboratory of the Department of Veterinary Medicine of the University of Perugia for analytical determination.

### 2.2. Microbial Determination

Samples were properly diluted with full-strength buffered peptone water (Oxoid Ltd., Basingstoke, UK) and homogenized (Stomacher 400 circulator, Seward Ltd., Norfolk, UK). Further serial decimal dilutions were performed with physiological saline sterile solution (Oxoid Ltd.), and the samples were plated in specific media for aerobic colony count (ACC), incubated under aerobic conditions at 30 °C for 72 h according to ISO 4833-1 using Plate Count Agar (PCA) (Oxoid Ltd.) [24] and *Enterobacteriaceae* (ENT) count, incubated under aerobic conditions at 37 °C for 24 h according to ISO 21528-2 using Violet Red Bile Glucose Agar (VRBG) (Oxoid Ltd.) [25]. For ENT a pure (uninoculated) VRBG agar layer was poured over the first bottom VRBG layer seeded with 1 mL of the appropriate sample dilution. The obtained counts were converted into log colony-forming units (CFU)/cm^2^. The same homogenized samples were kept for one day at 37 °C to isolate *Salmonella* spp. according to ISO 6579-1 [26]. Isolated strains were sent to the laboratory of the Istituto Zooprofilattico Sperimentale (Perugia, Italy) for identification. The strains were incubated at 37 °C for 24 h in trypticase soy agar (TSA) (Oxoid Ltd.) and serotyped according to the White–Kauffmann–LeMinor scheme by performing a slide agglutination test with polyvalent agglutination antisera against somatic and flagellar antigens (SSI diagnostics, Hillerod, Denmark).

### 2.3. Statistical Analysis

The data for ACC and ENT were submitted to separate one-way ANOVAs, by using each of the variables in Table 1 as the explanatory factor (environmental conditions, animal characteristics, hunting conditions and storage of the carcasses). Post hoc Tukey HSD tests were used to compare the least square means, by using the ‘emmeans’ package in the R statistical software (R Core Team 2021). Subsequently, both for ACC and ENT, a multi-way additive ANOVA model was built in a stepwise forward fashion for the factors which showed significance or a trend in the previously reported one-way ANOVA analyses. Explanatory factors were selected based on the Akaike Information Criterion (AIC) [27] in order to discover which variables produced the most relevant effect on the response variables. The interaction between these factors was therefore analyzed. For *Salmonella* spp., the prevalence and confidence interval (CI, 95%) were calculated, but no further analysis was possible, as the number of positive samples was not enough to allow correlation with the considered factors. 

## 3. Results

The samples were obtained from all 120 wild boars, as none received multiple or abdominal shots or were affected by severe pathology based on organ inspection, and there were no ruptures of the gastrointestinal tract during evisceration. The average values of the recorded data during hunting are reported in Table 2. 

All subjects were recovered within 60 min from the shot, bled out in the field within 30 min from recovery, eviscerated within 5 h, and promptly refrigerated. 

**Figure 2:**
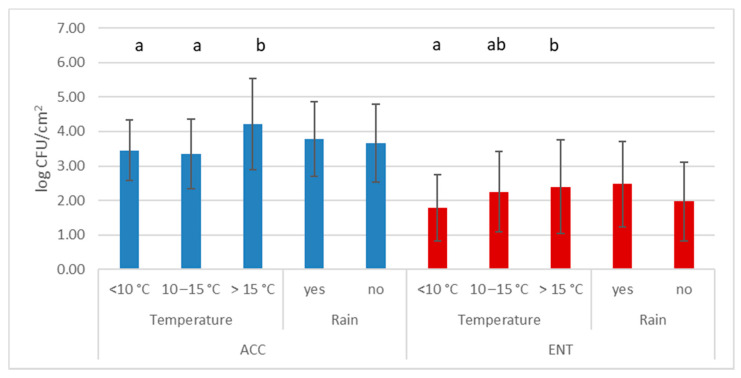
Mean values and standard deviation (bars) of aerobic colony count (ACC) and *Enterobacteriaceae* count (ENT) relative to environmental conditions during hunting; *n* = 120. Different superscript letters for factors indicate statistically different mean values (*p* < 0.05).

**Figure 3:**
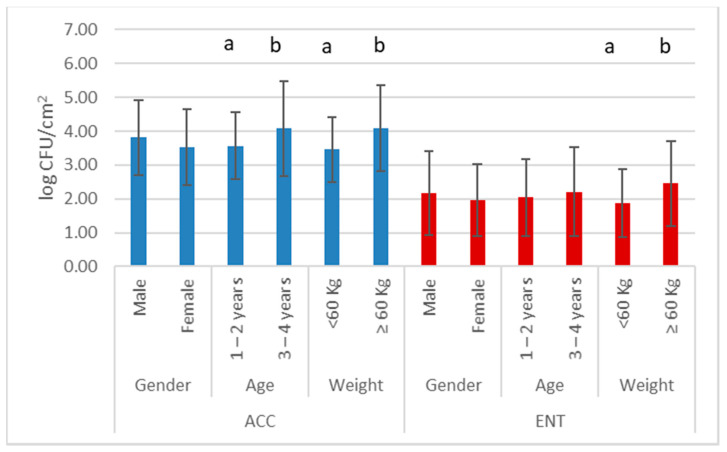
Mean values and standard deviation (bars) of aerobic colony count (ACC) and *Enterobacteriaceae* count (ENT) relative to hunted wild boar characteristics; *n* = 120. Different superscript letters for factors indicate statistically different mean values (*p* < 0.05).

The average values for ACC and ENT were 3.68 log CFU/cm^2^ (standard deviation 1.12) and 2.08 log CFU/cm^2^ (standard deviation 1.17), respectively. The results of the effects of environmental conditions on carcass hygiene are reported in Figure 2, and animal conditions (gender, age, and weights) are reported in Figure 3.

Although no differences were observed based on the presence of rain during the hunt, a trend was noted for ENT (*p* = 0.054) but not for ACC (*p* = 0.61). A difference between age classes was observed only for ACC.

The mean values of ACC and ENT relative to the hunting conditions are reported in Table 3. No effects were detected for the hunting method, position where the animal was shot, or elapsed time between shooting and evisceration. A trend of increased ENT was detected when wild boars were eviscerated 2 h after shooting.

**Figure 4:**
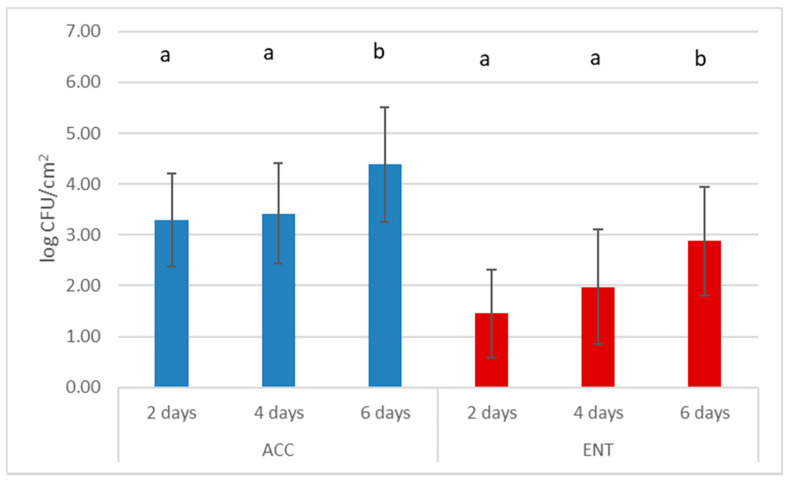
Mean values and standard deviation (bars) of aerobic colony count (ACC) and *Enterobacteriaceae* count (ENT) relative to storage time in collection center; *n* = 120. Different superscript letters for factors indicate statistically different mean values (*p* < 0.05).

Differences were registered for ACC and ENT when carcasses were stored in refrigerated conditions at the collection center for more than 4 days (Figure 4).

The multi-way analysis of the factors showed that the main factor affecting ACC was time of storage before skinning in the collection center (AIC = 5.57), followed by environmental temperature (AIC = 17.41) and wild boar weight (AIC = 20.05). The other factors were negligible. Regarding ENT, the main factor involved in increased values was time of storage before skinning (AIC = 6.97), followed by environmental temperature (AIC = 35.65), rainy days during hunting (AIC = 37.09), and time between shooting and evisceration (AIC = 37.79). Wild boar weight, which shows a significant difference between classes for ENT, was considered a negligible factor in the step-forward multi-way analysis.

Significant interactions were found between storage time and environmental temperature (*p* < 0.001) for both ACC and ENT, with higher loads on carcasses obtained on hotter days and stored for longer time, and, for ENT only, between storage time and time between shooting and evisceration (*p* < 0.05), with lower values when the time was shorter for both factors.

*Salmonella* spp. were isolated in 3 out of 120 samples, with a prevalence of 2.5% (CI 95% = 1.72–3.27%). One serotype detected was *Salmonella* Typhimurium and the other two were *Salmonella* Stanleyville.

## 4. Discussion

The results highlight that hygiene for game meat depends on several factors that are related to environmental and animal conditions, as well as hunter choices and handling [21]. Even processing procedures performed at game-handling establishments and cutting plants have to be considered and could be influenced by proper management of hunted animals in the harvest phase, as microbial populations tend to grow quickly when there are high loads in the early stage [20,28]. For this reason, maintaining a relatively low level of microbial contamination in the harvest phase is of paramount importance. 

The average values of ACC and ENT are similar to those reported by other authors [17,29] but lower than those reported by Avagnina et al., Russo et al., and Peruzy et al. (respectively in the Alps, northern Italy; the Tuscany region, central Italy; the Campania region, southern Italy) [16,18,30] and Mirceta et al. (in Serbia) [19]. The presence of a nearby collection center and proper training of the hunter and management of hunted wild boar could have influenced the lower average loads compared to the other studies. The choice of a well-trained hunter for the season and the hunting method has to be considered important, as the wild boars were not shot in multiple sites or in the abdomen, which lead to a higher probability of contamination due to gastrointestinal perforation [31]. The choice of non-collective hunting without free dogs that scare or attack wild boars and force them toward hunters (“braccata”), which is very popular in Italy [32], was made to avoid multiple shots and stress to the animals, reduce the time between shooting and refrigeration, and prevent the dogs from injuring the meat [33]. The “braccata” methods could therefore negatively affect the carcass hygiene and must be considered with caution in a certified game meat valuable chain.

Furthermore, the ACC and ENT levels are in line with those reported for “farmed” animals (bovine and swine) [34,35] and values suggested as acceptable by EU legislation criteria for hygiene processes (acceptable levels in swine between 4.0 and 5.0 log CFU/cm^2^ for ACC and between 2.0 and 3.0 log CFU/cm^2^ for ENT) [34]. Indeed, 6 and 12 samples out of 120 sampled carcasses (7.2% and 14.4% prevalence) exceeded the acceptable level set by EU legislation for ACC and ENT respectively [36]. Nonetheless, the hygienic criteria have to be considered not for individual carcasses, but for the whole process during production days (five carcasses sampled at random during each sampling session at the slaughterhouse) [35]. Taking this into consideration, under the hunting conditions reported, average to good microbial loads could be found even for hunted game meat.

The results from the multi-way analysis of the factors reveal that the main factor affecting both ACC and ENT was the number of days of refrigeration at the collection center, when animals are stored without skinning after evisceration. Collection centers are fundamental to provide quick evisceration and refrigeration of hunted wild boar, when game-handling establishments are far away or closed during the time of the hunt. Proper management of this step is crucial in order to avoid overload and increased temperature in the refrigerator [37]. Carcasses have to be sent to a game-handling establishment for the rest of the procedures, preferably within 4 days [21]. 

Another important factor to analyze is the environmental temperature, even if animals are sent to the collection center relatively quickly (always under 5 h from shooting). The effect of temperature has also been highlighted by other authors [17], showing that it is possible for the body temperature to be reduced quickly when the environmental temperature is <10 °C, thereby limiting microbial growth (for both ACC and ENT). Furthermore, a trend was seen with rainy days (the third most important factor for ENT), which provide better conditions for fecal or ground contamination or the spread of pre-existing contamination of the skin. Choosing the proper day for selective hunting is, therefore, important in order to ensure better hygiene for the carcasses. Unfortunately, the selective hunting season for wild boar is open in Italy even in late spring and summer, when the temperature is generally over 20 °C. In this situation, other harvest factors that can reduce the animals’ temperature have to be considered with caution (e.g., prompt evisceration in the field, isothermal refrigerated boxes for animal transport) [19].

Animal characteristics are another factor that can affect the final microbial load, mainly for ACC, as animals heavier than 60 kg have a higher load than lighter ones [17,20]. For ENT, although significant in the one-factor analysis, this aspect is somehow related to the time between shooting and evisceration and the days in the collection center, and results are negligible relative to these factors by multi-way ANOVA. Generally, heavier animals are difficult to manage in the forest, especially by single hunters, and therefore require more time and effort to pick up and transport to the collection center. For this reason, in a certified game meat chain, wild boar under 60 kg could be preferably selected. 

The last effect is the time between shooting and evisceration in the collection center for ENT, although this was not significant in the single ANOVA analysis. The influence of this factor has been reported by other authors [16,38] and was also observed in this survey combined with storage time. The maximum time for this procedure was less than 5 h; 82 out of 120 wild boars were processed in the collection center within 2 h, and no intestinal ruptures during evisceration occurred. The possible transposition of enteric bacteria from the intestines to the carcass caused by delayed evisceration is, however, controversial [39].

The statistical analysis revealed that the other factors were negligible, and no differences were found for ACC and ENT according to the hunting method adopted, showing that both techniques, if properly implemented, could achieve good carcass hygiene. No effects were found for shooting position, even if the probability of intestinal damage cannot be ignored when thorax is hit, as confirmed by the limited differences noted by other authors even when wild boars were shot in the abdomen [16,19]. 

Regarding isolation of *Salmonella* spp., the prevalence observed in this study is in line with or even lower that that reported by other authors [16,17,18]. Taking into account the hygienic criteria set by legislation [36], the number of positive samples relative to the sample population is below the set limit. *Salmonella* Tiphymurium and *Salmonella* Stanleyville were detected on three carcasses that had ENT values between 2 and 3 log CFU/cm^2^, highlighting that the presence of *Salmonella* spp. may not be related to ENT counts, as reported in swine [40]. The serotypes detected on the carcasses were reported in hunted wild boar feces, which have a wide range of serotypes [32], but few studies have reported the most prevalent serotype found in the carcasses [41,42]. To lower the prevalence of *Salmonella* spp. in “farmed” game meat, an integrated approach is suggested, with the application of indicators of specific harmonized epidemiological indices [43]. Further studies are therefore needed to define whether, in a “forest to fork” approach, similar indices could be implemented for certified wild boar meat to reduce the levels of *Salmonella* spp. in the meat. 

## 5. Conclusions

This survey supports the concept that by using specific hunting techniques, ensuring that hunters are properly trained, selecting the proper days for hunting (<10 °C without rain), selecting the animals to shoot (healthy animals < 60 kg in weight), promptly transferring wild boars to a collection center for evisceration (<2 h), and storing carcasses under refrigeration in the collection center for a short time (<6 days), it is possible to achieve better hygiene during the harvesting phase of wild boar meat. The collection center also plays a crucial role in proper evisceration of the animal, in order to avoid intestinal ruptures and, therefore, contamination of the carcass. Furthermore, the prevalence of *Salmonella* spp. is low, even if further improvements could be made to control these pathogens in the game meat chain.

## Figures and Tables

**Table 1:** Distribution of samples according to the hunting questionnaire provided.

Factors and Classes	Number of Samples
**Environmental temperature (°C)**	
<10	58
10–15	23
>15	39
**Rain**	
Yes	95
No	25
**Animal weight after shooting (kg)**	
<60	77
≥60	43
**Animal age (years)**	
1–2	92
3–4	28
**Animal gender**	
Male	70
Female	50
**Hunting system**	
Aspetto	69
Girata	51
**Shooting site**	
Head or neck	52
Heart or shoulder	33
Thorax	35
**Time between shooting and evisceration (hours)**	
<2	82
2–5	38
**Time in storage before skinning (days)**	
2	44
4	37
6	39

**Table 2:** Mean values for environmental temperature and wild boar characteristics.

Factors and Classes	Mean Value ± Standard Deviation
Environmental temperature (°C)	11.61 ± 6.34
Animal age (years)	1.96 ± 0.91
Animal weight (kg)	51.13 ± 24.15

**Table 3:** Mean values of aerobic colony count (ACC) and *Enterobacteriaceae* count (ENT) relative to hunting methods and characteristics (in log CFU/cm^2^; *n* = 120).

**Hunting Methods**						
	Aspetto		Girata		SEM	*p*-value
ACC	3.66		3.79		0.101	0.854
ENT	2.01		2.17		0.104	0.452
**Shooting Position**						
	Head/neck	Shoulder/heart		Thorax	SEM	*p*-value
ACC	3.62	3.55		3.90	0.102	0.382
ENT	1.95	2.08		2.26	0.099	0.494
**Time between Shooting and Evisceration**						
	<2 h		2–5 h		SEM	*p*-value
ACC	3.64		3.77		0.104	0.565
ENT	1.95		2.35		0.107	0.082

## Data Availability

The datasets generated for this study are available on request from the corresponding author.
